# One-Step Laser Patterned Highly Uniform Reduced Graphene Oxide Thin Films for Circuit-Enabled Tattoo and Flexible Humidity Sensor Application

**DOI:** 10.3390/s18061857

**Published:** 2018-06-06

**Authors:** Rowoon Park, Hyesu Kim, Saifullah Lone, Sangheon Jeon, Young Woo Kwon, Bosung Shin, Suck Won Hong

**Affiliations:** 1 Department of Cogno-Mechatronics Engineering, Department of Optics and Mechatronics Engineering, College of Nanoscience and Nanotechnology, Pusan National University, Busan 46241, Korea; parkrowoon@naver.com (R.P.); deerbread@naver.com (H.K.); saifullah.lone@gmail.com (S.L.); jsfhse@naver.com (S.J.); 2 Department of Nano-Fusion Technology, College of Nanoscience and Nanotechnology, Pusan National University, Busan 46241, Korea; handmade4522@gmail.com

**Keywords:** graphene oxide, self-assembly, laser-exposure, circuit, humidity sensor

## Abstract

The conversion of graphene oxide (GO) into reduced graphene oxide (rGO) is imperative for the electronic device applications of graphene-based materials. Efficient and cost-effective fabrication of highly uniform GO films and the successive reduction into rGO on a large area is still a cumbersome task through conventional protocols. Improved film casting of GO sheets on a polymeric substrate with quick and green reduction processes has a potential that may establish a path to the practical flexible electronics. Herein, we report a facile deposition process of GO on flexible polymer substrates to create highly uniform thin films over a large area by a flow-enabled self-assembly approach. The self-assembly of GO sheets was successfully performed by dragging the trapped solution of GO in confined geometry, which consisted of an upper stationary blade and a lower moving substrate on a motorized translational stage. The prepared GO thin films could be selectively reduced and facilitated from the simple laser direct writing process for programmable circuit printing with the desired configuration and less sample damage due to the non-contact mode operation without the use of photolithography, toxic chemistry, or high-temperature reduction methods. Furthermore, two different modes of the laser operating system for the reduction of GO films turned out to be valuable for the construction of novel graphene-based high-throughput electrical circuit boards compatible with integrating electronic module chips and flexible humidity sensors.

## 1. Introduction

Graphene is a two-dimensional (2D) monolayer of sp^2^ carbon atoms arranged in a hexagonal or honeycomb lattice. In the previous two decades, the pioneering work on the isolation and characterization of pristine graphene with remarkable high charge-carrier mobility paved a way to the rise of intense graphene research [1]. Generally, graphite is composed of the staked graphene-sheets in a Bernal (ABA) or Rhombohedral (ABC) sequence, with the layers kept together by weak van der Waals forces [2]. After the initial discovery, many researches have been focused on the artificial synthesis of graphene on a large scale, rather than the micromechanical exfoliation method [3]. The growth techniques developed to produce highly qualified graphene include thermal chemical vapor deposition [4], plasma enhanced chemical vapor deposition [5], chemical exfoliation and reduction from graphite [6], thermal decomposition of SiC [7], mechanical milling exfoliation [8], and unzipping of carbon nanotubes [9]. Among these main graphene production techniques, the chemical exfoliation of graphite using strong oxidants has been intensely explored to prepare colloidal suspensions of individual 2D graphene oxide (GO) sheets dispersed in a volatile solvent, followed by the reduction of GO, which yields reduced graphene oxide (rGO) [10,11].

GO is a versatile material, which consists of atomically thin hydrophilic sheets of graphene decorated with various oxygen-containing functionalities (mainly epoxide, hydroxyl, carbonyl, and carboxyl groups) on their basal-planes and edges; in detail, the epoxide and hydroxyl groups (primary components) are located on the basal-plane of the GO, whereas, the carbonyl and carboxyl groups (minor components) are distributed at the edges of the GO [12,13,14,15]. Due to the tunable features of the structural defects and oxygen functional groups, electrically insulated GO can readily be converted to conducting rGO for the broad applications that include transparent conductive films [16,17], field-effect transistors [18,19], chemical and biological sensors [20,21], photovoltaics [22,23], acoustic actuators [24], and thermal interface materials [25].

To expand suitable applications in these fields, various techniques have been developed to organize colloidal GO on a substrate, minimizing susceptible external conditions during the deposition process, such as vacuum filtration, flow-assisted casting, Langmuir-Blodgett assembly, ink-jet printing, spraying, and drop-casting [26,27,28,29,30,31]. In this context, a few strategies have recently demonstrated the effective manipulation of the controlled evaporative self-assembly process to produce highly ordered GO structures via irreversible solvent evaporation from a colloidal solution. In addition to this GO thin film assembly process, the effective reduction process of GO to prepare graphene derivatives (i.e., rGO) remains a significant interest and has been performed by several methods using simple chemical or thermal reactions, photoreduction, and microorganism-mediated reduction [27,32,33]. In addition, conventional lithography or soft-lithography have also been tested to organize rGO on substrates and its micropatterning into useful hierarchically ordered structures [34,35]. However, most of the aforementioned approaches involve multiple procedures and are time-consuming system or are unsuitable for large-scale production. Thus, the attention towards the effective strategies for the reduction of GO without the use of toxic chemicals or high temperature is highly desired, along with the spontaneous patterning process for the potential to scale-up production.

Herein, we report a facile fabrication method for the direct writing of high-performance rGO-based printed circuits and humidity sensing devices, with a flexible format, by the laser-scribing operation of self-assembled GO films on polymer substrates. We highlight two important aspects that may assist to the scale-up design of the GO thin film deposition (i.e., self-assembly process) and synergistically diversify the laser-scribed reduction and the micropatterning of GO thin films, regarding rapidity and simultaneity. Firstly, flow-enabled self-assembly (FESA) suggests a simple and viable route to spontaneously assemble the suspended GO sheets into highly uniform GO films over a large area on flexible polymer substrates at room temperature [36,37]. The FESA process has garnered great interest in fabricating ordered micro- or nanostructures in multiscale (e.g., thin films and patterns) using various types of dispersions in a precisely controllable manner [38,39,40,41,42]. In the present work, the FESA process involves the loading of an aqueous dispersion of GO sheets in-between the confined geometry, composed of an upper stationary top-blade (e.g., slide glass) over a lower receiving substrate placed on a motorized moving translational stage. In addition, the use of laser irradiation on the deposited GO thin films has garnered much attention for the simple GO reduction process; the conversion levels of GO into rGO could be varied by a direct or indirect laser exposure condition as reported previously [43,44]. For example, the direct laser exposure (i.e., in-plane) results in the complete reduction of GO, showing the high conductivity on the patterned rGO films for the construction of flexible electronic circuits. Hence, in the case of the indirect laser exposure through the back plane of a polymer film, fewer reduced GO films are formed in the patterned surface region bearing relatively rich oxygen groups, which can be extended for the useful applications. We demonstrated selectively patterned laser-scribed rGO films on a plastic substrate for the use of graphene-based flexible printed circuit boards compatible with integrated electronic module chips, and the rGO patterned arrays were facilitated as humidity sensors and heating modules as lightweight wearable electronics. Our present work may provide an extremely simple cost-effective way to produce a prototype of the electronic tattoos mounted on the skin or even engraved directly on the surface of polymeric films along with other existing semiconductor fabrication technologies [45].

## 2. Materials and Methods

### 2.1. Highly Uniform Thin GO-Film Fabrication via FESA Process on Polymer Substrate

**Figure 1:**
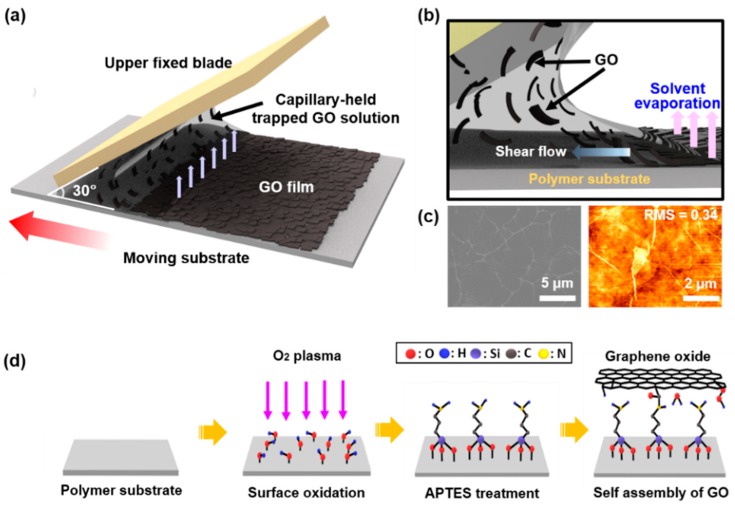
Fabrication process of the uniformly deposited GO thin film on a polymer substrate via flow-enabled self-assembly. (**a**) Schematic illustration of the self-assembly of GO sheets by dragging the trapped GO colloidal solution in restricted geometry consisted of an upper tilted stationary blade and a lower moving substrate on a motorized translational stage. (**b**) Schematic side-view illustrates the evaporative shear flow induced deposition of GO sheets at the end of the meniscus on a flat substrate. (**c**) Scanning Electron Microscope (SEM) image (left) and Atom Force Microscope (AFM) image (right) of the highly uniform GO thin film formed on a substrate. (**d**) Schematic steps for the surface modification of polymer substrate (i.e., PP) using mild oxygen plasma and the successive self-assembled monolayer treatment of APTES.

GO solution was prepared using the modified Hummer method, as described in our previous report [46]; the typical concentration of GO was appropriately set as 2 mg/mL, dispersed in deionized (D.I.) water for the uniform deposition via the FESA process on a flat polypropylene substrate (PP, t = 40 μm) as illustrated in Figure 1a. The PP substrate was cut in the size of 4.5 cm × 5.5 cm, and it was firmly placed on the motorized translational stage as a lower substrate. To avoid the warpage or delamination from the bottom stage, we used double-sided tape. Next, the upper blade (i.e., slide glass) was adjusted at 30 degrees, using a multi-axis-support right above the flat PP substrate at a distance of ~100 µm. The as-prepared colloidal solution of GO (~50 μL) was injected in confined geometry (i.e., upper fixed blade and flat PP substrate), thus the capillary-held trapped meniscus could be formed. Finally, the translation stage traveled at a constant speed of 15 mm/s and deposition number of 100 cycles to coat the surface of PP film.

### 2.2. Surface Modification of PP Film and Self-Assembled APTES-GO Sheet

The surface of the hydrophobic polymeric substrate was modified with the self-assembled monolayer (SAM) to enhance the adhesion between GO sheets and PP. Prior to performing the FESA process, the PP substrate was thoroughly washed with acetone, ethanol, D.I. water, and isopropyl alcohol (IPA) to remove the presence of any contaminants. The surface of PP substrate was then hydrophilicized with mild O_2_ plasma (50 W, 50 sccm, 30 s). After the exposure of plasma, the PP films were immediately immersed into 3-aminopropyltriethoxysilane solution (APTES, 99% purity, Sigma, St. Louis, MO, USA) with a volume ratio of 5:1 in the mixture of acetone and D.I. water for 1 h at room temperature; terminal amine groups were covalently bound to the PP surface. The silane residues were rinsed away with acetone and then blown with nitrogen gas.

### 2.3. Characterization and Measurements

The surface morphology of FESA-GO films and laser-scribed patterned rGO were observed by optical microscopy (OM) and scanning electron microscopy (SEM, SUPRA40VP). The surface roughness of these samples was also measured by atomic force microscopy (AFM, Park Systems, Suwon, Korea). Raman spectra and Raman mapping images were collected by Raman spectroscopy with 532 nm laser excitation (UniNanoTech Co., Yong-In, Korea). Packaged light emitting diode, LED (1.6 mm × 0.8 mm, SMD type, Kingbright, Shenzhen, China) were mounted on the surface of the laser-scribed patterned circuits using silver paste, and the electrical property measurements were carried out in ambient condition using semiconductor parameter analyzer (Agilent 4156A). The change in capacity of graphene-based humidity sensor crafted by indirect laser irradiation during the periodic exposure of moisture was detected using an LCR meter (Gwinstek, LCR-916, New Taipei City, Taiwan) at 100 kHz. To examine the heat-distribution for the patterned arrays of indirect laser-scribed patterned rGO, the temperature changes modulated by the applied voltage were imaged by visual IR thermometer (Fluke VT04).

## 3. Results and Discussion

Figure 1 illustrates a schematic of a sequential process to craft a highly uniform thin film of GO on APTES-treated PP substrate via the FESA process. The first step was to create a restricted geometry that constrain the GO suspension by placing a drop of GO aqueous solution between the upper blade (positioned at a constant angle, θ = 30°) and the lower flat APTES-treated polymer substrate with a fixed gap (~100 µm); the capillary-held trapped meniscus was formed (Figure 1a). Next, the lower substrate (i.e., APTES-treated PP) was moved repetitively back-and-forth by a motor-driven translation stage to drag the entrapped GO meniscus by capillary force between the two substrates and deposit GO sheets. The dragged meniscus left behind GO sheets uniformly by the repetitive motion at a constant speed of 15 mm/s and deposition number of 100 cycles. In this process, the evaporation rate was highest at the edges of the solution drop. In particular, the gradient in evaporation rate at the edge of a capillary-held solution drove the individual GO sheets under shear-flow with a balanced surface tension towards the continuously evaporating contact line. In this way, this regular deposition of GO sheets resulted in a uniform ultrathin GO film (~40 nm) on PP substrate (Figure 1b). The surface of the casted GO film by the FESA process was explored by SEM (left side) and AFM (right side) as shown in Figure 1c. On the other hand, to enhance the adhesion between the polymer substrate and GO film, we modified the surface of PP substrate using mild oxygen plasma and subsequent SAM treatment (i.e., APTES), as schematically illustrated in Figure 1d [47]. As a result, the depositing GO sheets were covalently bonded to the terminal amine groups formed on PP substrate. In addition, the resultant tight intermolecular interaction with van der Waals forces between the GO sheets was fairly identified with the extremely low surface roughness (RMS = 0.34 nm) as measured using AFM.

**Figure 2:**
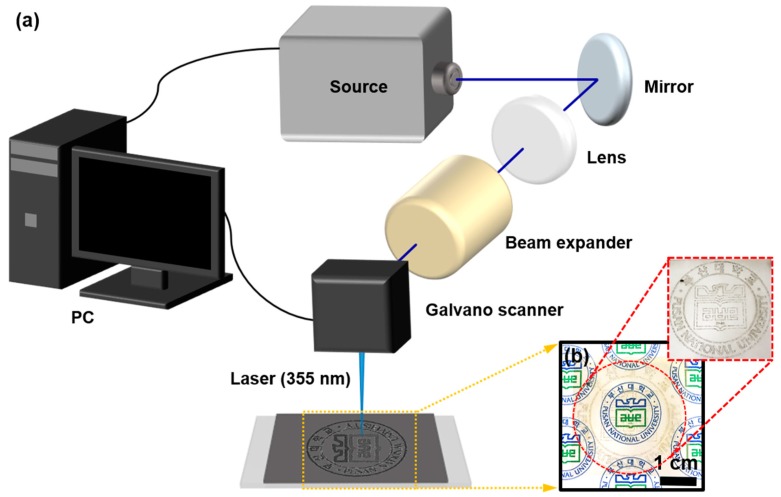
(**a**) Schematic illustration of UV pulse laser system for the reduction of GO. (**b**) Digital image of university logo with sharp contrast (inset) engraved by laser direct writing (LDW) on GO coated PP substrate, showing optical properties with see-through type transparency.

A simple laser setup was used to reduce GO thin film into the patterned rGO on PP substrate as shown in Figure 2. The Nd:YVO4 UV pulsed laser (*λ* = 355 nm) system in TEM_00_ mode was utilized for GO reduction process (Figure 2a). In our system, the pulse duration, at a repetition rate of 30 kHz, was 20 ns, and the laser beam diameter was set as 1.5 mm. By tuning the laser intensity distribution with the fluence of 0.5–1 J/cm^2^, the GO reduction was performed between the lowest (0.04 W) to maximum (0.8 W) power gap; the specifications of the laser system are described in Supporting Information (Figure S2). Digital photograph in Figure 2b portrays the university logo (PNU) which was fabricated on laser-scribed GO-coated PP substrate showing the discrete and sophisticated patterns (i.e., GO-rGO-GO) that can be constructed using this simple one-step laser system. This laser direct writing (LDW) method presented in this work has several advantages over conventional photolithography for the pattern transfer process at the micron scale without the use of complicated processes such as spin-casting photoresist, UV exposure, development, and photoresist removal. Therefore, LDW is easy to carry out and achieves the end results, desired patterning and the conversion GO to rGO simultaneously, in a quick and facile manner. Generally, the possible mechanism for the laser-induced reduction of GO to rGO can be explained in light of photochemical and photothermal reactions [48]. It is important to note that the photochemical reduction operates in the presence of sacrificial compounds (e.g., photocatalysts and gasses). Whereas photothermal reduction under ambient conditions is induced by UV laser irradiation, which engages with the localized heat generation to cause the deoxygenation of GO [49]. GO is composed of sp^2^ and sp^3^ hybridized carbon networks bonded to oxygen functional groups to behave as an insulator [13]. However, the electrical excitation effect in GO generated by the focused UV irradiation can selectively reduce the GO by a spontaneously-generated thermal effect [48]. In fact, the UV laser excites single photons in the graphene domain from the valence to the conduction band, the electron excitation of GO requires a threshold photon energy of at least 3.2 eV [50]. However, the energy (E~3.49 eV) provided by the laser irradiation in photoreduction is much higher than the threshold photon energy to excite an electron from valence to the conduction band [51]. Therefore, the strong electron-hole coupling within GO sheet causes local heat generation, at which the exceeded temperature decomposes the oxygen groups, and the dissociation of oxygen groups with different binding energies (O-H (0.7 eV)/C-O-C (1.9–2.1 eV)/C=O, COOH (3–4 eV)) increases [52]. Consequently, the removal of oxygen functional groups in the form of CO, CO_2_, and H_2_O, and the thermally assisted structural rearrangement (i.e., the transformation of sp^3^ to sp^2^ conjugated hexagonal structure in carbon lattice) contribute to the reduction of GO into rGO [48,49,50,51].

**Figure 3:**
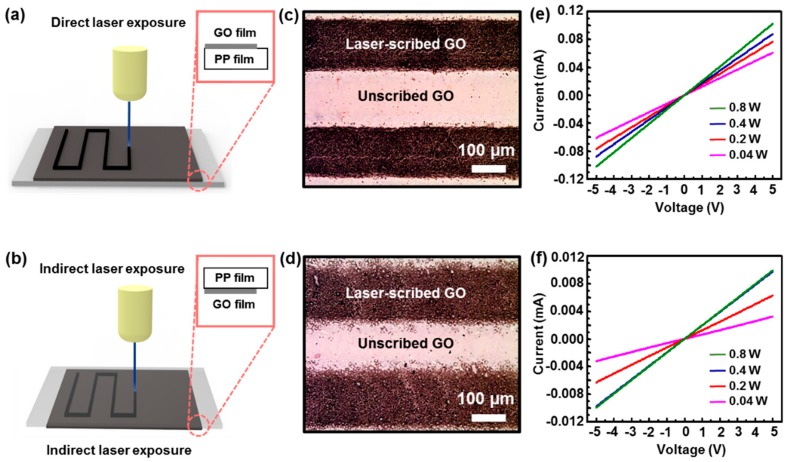
Laser-scribed patterning and reduction of GO thin films formed on PP substrate to fabricate rGO-GO-rGO stripes with a controlled mode of operation. (**a**) Direct laser exposure on GO-PP substrate. (**b**) Indirect laser exposure on GO film through PP barrier. The inset drawings in figure (**a**,**b**) illustrates the surface position of GO film and PP substrate against the laser exposure. (**c**,**d**) Optical micrographs showing the patterned rGO-GO-rGO stripes operated by direct and indirect laser exposure (dark lines: laser-scribed rGO, bright line: unscribed pristine GO). (**e**,**f**) Typical *I-V* characteristics of the samples scribed by direct and indirect laser operations.

The laser-scribed reduction and the micropatterning of the GO film on PP could be achieved through two different routes, as summarized in Figure 3. The direct laser operation (i.e., in-plane) for patterning with a line width of ~180 μm, and the line/space of ~200 μm ensured the complete reduction of GO to rGO (Figure 3a), whereas, the indirect irradiation through a PP *barrier* resulted in partially reduced GO, as shown in Figure 3b. The conversion of GO to rGO was first observed by OM, a color distinction was clearly observed between the laser-scribed rGO (dark stripe) and unscribed (light stripe) GO film on PP substrate (Figure 3c,d). In addition, the direct and indirect-laser scribed GO films were clearly noticeable as compared in Figure 3c,d; the former was displayed by deep dark stripes, while the latter was represented in a lighter color. In detail, the surface of direct laser-scribed rGO regions was confirmed to have uniformly swollen morphologies with a sharp contrast at the boundaries of GO-rGO-GO. Hence, the indirectly exposed regions contained structural defects similar to the surface crater and showed more-or-less uneven contrast at the edges of line patterns (Figure 3d). In particular, PP film played an important role as an intermediate barrier when the laser reached the GO film, which affected the final surface morphologies of the patterned rGO film. It is interesting to note that the substantial absorption inside PP film did not occur when the laser was exposed, since the transmittance of PP film used in this experiment was 99% at 355 nm wavelength. Moreover, the physical and chemical decomposition in PP film could not be developed by the low absorption coefficient [53]. Instead, the UV laser beam passing through the PP film (Figure 3b) induced a series of interactions electrical excitation and thermal effects at GO surface, and the resulting thermal shock was propagated from GO film to the PP film [48]. Rapid thermal conduction due to trapped heat between the GO and PP films resulted in the transform of the PP film, that is, simultaneous phase change and viscosity decreased through molecular dissociation. This phase change affected the crystallinity and deformation of the PP film, and the slight deformation of the PP film influenced the surface morphologies of the GO reduced by the laser irradiation [54,55].

**Figure 4:**
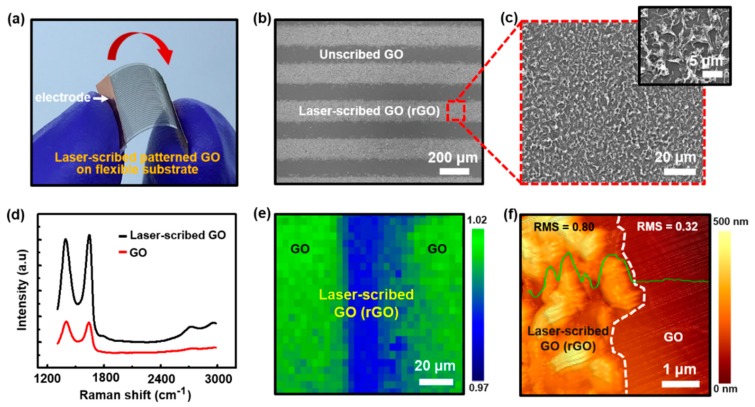
(**a**) Digital image of laser-scribed rGO patterned arrays connected to copper electrodes on a flexible PP substrate. (**b**) Macroscopic SEM image of the laser-scribed GO film (bright lines: laser-scribed rGO, black lines: unscribed pristine GO). (**c**) SEM image of the close-up surface area in (**b**) with swollen microstructural hierarchies. (**d**) Raman spectra of laser-scribed GO (black) and GO (red) indicate the reduction of GO film in the range of 1300−3000 cm^−1^. (**e**) Raman mapping image of GO-rGO-GO region. (**f**) AFM image at the boundary between the laser-scribed rGO and pristine GO film marked by dotted white line; green line shows relative height profile, ~500 nm.

To confirm the successful reduction and patterning of the insulating GO film by two different laser-scribing methods; electrical tests were performed separately on the patterned rGO films fabricated by direct and indirect laser exposures. The graphs in Figure 3e,f demonstrates the typical *I-V* characteristics of the samples with two electrodes with a voltage range of −5 to 5 V (laser power range of 0.04–0.8 W, laser speed of 100 mm/s, and fluence of 0.5–1 J/cm^2^), designed from the samples scribed by direct and indirect laser exposures. Current values of the individual lines, measured with two electrical contacts at both ends, showed significant differences between the samples of the laser-reduced graphene using different laser-scribing methods. It is well known that the current of rGO film prepared by laser irradiation depends on the amount of eliminated oxygen-containing group from the GO basal plane. The *I-V* curves in Figure 3e,f revealed that both the direct and the indirect laser exposures would yield patterned rGO films with varied intensities. The surface morphology of patterned GO film by direct laser-scribing method is highlighted in the Figure 4. Figure 4a shows a digital photograph of the stripe patterns by the direct laser-scribed GO film connected to copper electrodes (i.e., two-terminal device) on a flexible PP substrate. To explore the surface morphology of this device, this sample was measured by SEM (Figure 4b). Similar to Figure 3c, the SEM image also clearly shows color distinction between the laser-scribed regions (i.e., rGO) and unscribed GO film. When the laser passed directly through the GO film, the rGO region yielded highly neat edges of the line patterns with a width of ~180 μm and spacing of ~200 μm, as shown in Supporting Information, Figure S3. Figure 4c also shows a magnified SEM image of the local area of rGO film with uniformly swollen microstructural hierarchies (Supporting Information, Figures S4 and S5, for the surfaced morphology according to indirect laser exposure).

To examine the reduction of GO thin film via laser ablation, the GO thin film on Si/SiO_2_ substrate was characterized by Raman spectroscopy with the local mapping images (Figure 4d). The Raman spectrum of GO thin film (red) displayed two bands, one at 1393 cm^−1^ (D band) and the other at 1645 cm^−1^ (G band). The G band is related to the vibration of sp^2^ carbon-double bonding in the carbon-based hexagonal structure; while the D band signifies the vibration of sp^3^ carbon-dangling bonding affected by the structural defects such as grain boundaries and vacancy of carbon atoms. The low D/G band intensity ratio of the rGO thin film (*I_D_/I_G_* = 0.97), compared to the GO thin film (*I_D_/I_G_* = 1.02) after laser irradiation, indicates that the structural defect in carbon lattice structure was partially restored to the π-bond conjugated hexagonal networks. As shown in Figure 4e, Raman mapping of the D and G bands at the laser-scribing GO film (i.e., GO-rGO-GO region) was clearly imaged; the closer to the green the pristine GO and the closer to the blue the laser-reduced GO. Furthermore, the morphology of a local interface between the laser-scribed GO-area (left region) and unscribed GO-area (right region) marked by a white dotted line is presented in Figure 4f. The flat GO film was transformed into a swollen and coarse rGO with a height of up to ~500 nm. The green solid line represents the relative height profile of the laser scribed and unscribed GO film with root-mean-square (RMS, R*_q_*) values (i.e., surface roughness) of 0.80 and 0.32 nm, respectively(Figure 4f).

**Figure 5:**
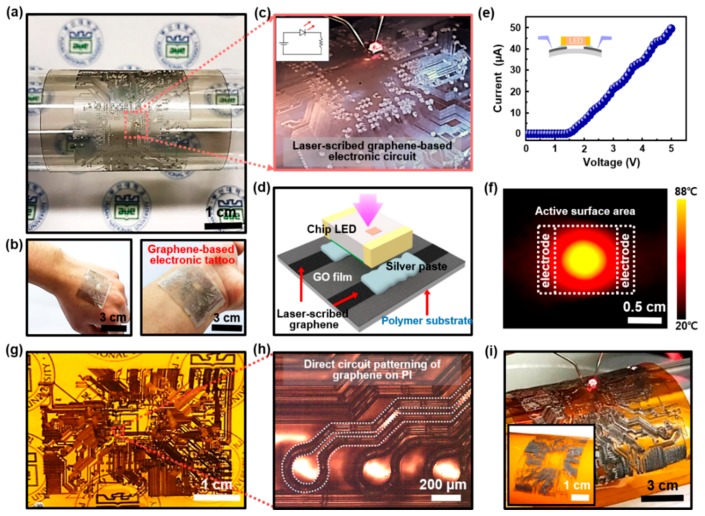
Graphene-based flexible electronic circuit boards produced via programmable direct laser-scribing. (**a**) Digital image of the representative flexible circuit board filled with the patterned rGO circuital lines, wrapped around a curved glass-tube surface. (**b**) Graphene-based electronic-tattoo attached on a wrist and back of a hand. (**c**) Light-up a surface-mounted LED integrated on an rGO-based circuit board in (**a**). (**d**) Schematic illustration of a chip LED bonded onto the isolated two terminal pads using silver-based solder paste. (**e**) Typical *I-V* characteristics of a chip LED integrated on the circuit board at bending state. (**f**) Thermographic image from a sticky plastic electronic tattoo (i.e., rGO-based microheater); thermal distribution ranged between 20 to 80 °C. (**g**) Digital image of the flexible circuit board prepared by direct laser-scribing of Kapton film. (**h**) Magnified OM image of a discretely patterned surface on the Kapton film; the white dotted lines indicate the laser-scribed conductive circuital lines. (**i**) Light-up a surface-mounted LED integrated on the two-terminal rGO circuit pads embedded on Kapton film.

To expand the application of micro-patterned rGO that was confirmed to have sufficient current flow by the successful laser-scribed reduction of GO, we generated large scale flexible microcircuit arrays with relatively complicated configurations in the designed drawing area, utilizing the advantage of the programmable laser direct writing. This was important because the patterned conductive films with specific structures in a flexible format can be applied to a variety of applications such as touch sensors, displays, solar cells, biosensors, and other possible unconventional electronics similar to the flexible printed circuit board (FPCB) [56,57,58,59,60]. In this study, we suggest a simple prototype of flexible circuit substrates composed of laser-scribed graphene formed on the polymeric materials. The designed scheme and procedures with the experimental results are highlighted in Figure 5. A graphene-based flexible circuit board was wrapped around a rounded glass-tube surface with a circuit full of somewhat complex patterns, showing optical properties with see-through type transparency as shown in Figure 5a; all of the fronts were covered with GO, and only the patterned arrays for the circuit construction were composed of laser-scribed rGO. Particularly, this circuit-enabled substrate was composed of a very thin polymeric film, which easily made a conformal contact with the textured surface of the skin when a slight amount of a biocompatible adhesive layer was provided (i.e., water-soluble polyvinylalcohol, PVA). Figure 5b shows a typical demonstration of a graphene-based *electronic tattoo* that displays a reliable mechanical stability when mounted on the human skin on the back of the hand (left), and wrist (right); the idea was visually conceptualized by mimicking the circuital tattoo engraved in the skin. This could be considered as a basic platform for the skin-integrated devices with highly conductive interconnections (e.g., electronic tattoo) for prototyping biometric tracking [61]. As illustrated in the Figure 5c,d, a packaged chip LED was used to test the compatibility of the surface-mount technology (SMT) by it placing directly onto the graphene-based FPCB; a silver-based solder paste provided a robust connection by filling the gap between the chip LED contacts and the isolated two terminal pads of laser-scribed rGO within the specific circuit area (Figure 5d). Figure 5e shows the representative *I-V* characteristics of a single LED undergoing the bending state (Figure 5c). The turn-on voltage of the LED was ~1.6 V when the applied voltage was swept 0 V to 5 V across the rGO interconnects of the circuit. It was confirmed that the higher the applied voltage, the larger the amount of emitted light, since the light emission of the LED was directly proportional to the applied voltage. At a different location in the microcircuit area, an array of parallel stripe patterns of rGO (i.e., individual narrow conductive channels) was separately tested by employing conductive copper electrodes that were directly connected to the probe station. Interestingly, the surface temperature of the electronic tattoo was modulated by the applied voltage at the ambient condition, as shown in Figure 5f. This thermographic image measured by visual IR thermometer clearly represents the temperature changes at the surface of the graphene-based electronic tattoo. Especially due to the electrical resistance in the rGO-film by indirect laser scribing route, the thermal distribution was laid in the range of 20 to 80 °C as measured. At the closer to the center area of the rGO film, the more gradually the temperature increased up to ~88 °C. Due to the wide range of the control over a temperature, sticky plastic electronic tattoos (i.e., instant hot rGO-based microheater) could be used for thermotherapy for practical healthcare applications [62].

In a similar way to the laser operating method presented in this study, some experiments have been reported obtaining graphene-based nanomaterials by direct laser-scribing of polymer films. Based on the prior studies [63,64,65,66], we have produced a flexible printed microcircuit board construction from a Kapton polymer sheet (PMDA-ODA; polyimide, PI) that contained condensations of oxydimethyl aniline and pyromellitic dianhydride, utilizing the aforementioned laser-scribing technique. However, the mechanism of the graphene generation was completely different from the GO precursor-derived method. Briefly, a small fraction of the C_aryl_-C bond appeared to be thermally activated under relatively high localized temperatures from UV laser irradiation, which resulted in a loss of cleaved fragments such as -C_6_H_4_N(CO)- and reduced content of nitrogen and carbonyl groups. Here, the aromatic repeat units in PI are known to provide a unique environment for the laser-induced graphitization in the polymeric lattice, as they own a flat structure in etheric-oxygen near the bridging part [63]. This is the reason why Kapton has high thermal stability and mechanical strength [63,64,65,66]. Unlike laser-scribed reduction of GO films crafted using FESA on PP substrate, the PI film can serve both as an insulating substrate and carbon-feeding material itself. Through the programmable laser direct writing system, it was possible to produce a directly-written in-plane graphene-based circuit board in the desired drawing area as shown in Figure 5g. The magnified optical micrograph is presented in Figure 5h to distinctly show the discrete and smooth patterned surface with a repetitive line-width of ~50 μm. In our system, the laser exposed carbonized regions (i.e., dark black area) were composed of a graphitic multilayer, thereby, the electrical current flowed only through the confined circuital surface channels. Using this featured sample, we tested the suitability for use as interconnects using a single chip LED installed by the same technique on the flexible PP circuit board (Figure 5d) that applied a voltage of 2 V through the two external electrodes as shown in Figure 5i. Uniform and constant emission characteristics were clearly observed without any significant deformation of the silver paste-on contact pads.

**Figure 6:**
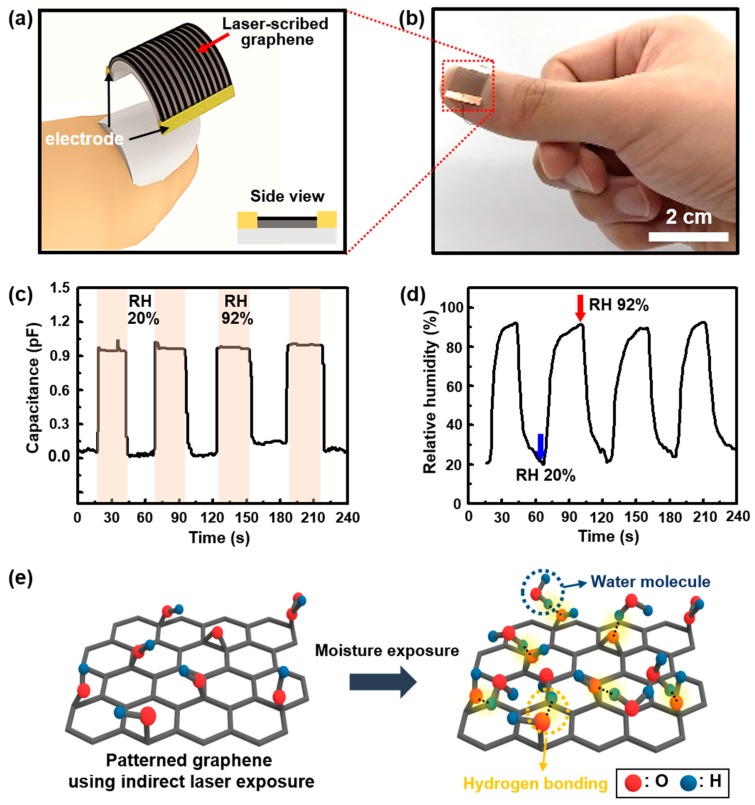
Schematic view (**a**) and digital image (**b**) of the laser-scribed rGO-based flexible humidity sensor attached on a nail (e.g., press-on nail wearable sensor), consisting of an indirect laser-scribed rGO film on flexible PP substrate integrated with a copper electrode. (**c**) The real-time signal responses as measured cyclic capacitance changes from the LDW GO-based humidity sensor, exposed to RH in the range of 20% to 92% at 30 s intervals. (**d**) The monitored RH changes collected from the real-time data logger correspond to the graph in (**c**). (**e**) Schematic drawing of absorption process of water molecules by hydrogen bonding on the partially reduced GO surface after moisture exposure.

For more practical applications, laser-scribed patterned rGO on a PP substrate was used as a flexible humidity sensor, such as an electronic tattoo attached on a nail (e.g., pre-glued press-on nails), for real-time monitoring of the environment, as schematically shown in Figure 6a,b. In this experiment, we used partially reduced GO films produced by indirect laser exposure as described in Figure 3d, since the indirect laser irradiated GO thin film contained abundant oxygen groups that acted as active channels, which provided the efficient adsorption sites of water molecules. In the case of stacked GO sheet layers with swollen characteristic features, due to the laser irradiation, the GO film had a high surface-to-volume ratio and a large density of surface vacancies. In addition, the oxygen functionalized groups, which are regarded as defective sites for lowering the conductivity of the graphene, could be applied to an environment more suitable for humidity sensing. Figure 6c shows the real-time signal responses as measured capacitance values from the LDW GO-based humidity sensor, exposed to varying relative humidity. The devices were characterized by the absorption and desorption of water molecules for a range from 20% to 92% RH during the response-recovery cycles at atmospheric pressure; the moisture-flow onto the devices was periodically engaged by the carful control of a humidifier at 30 s intervals. Figure 6d clearly represents the changes of RH levels that were collected from real-time log-data (HOBO Data Logger, U14), which exactly corresponded to the capacitive humidity sensor. The clear changes of capacitance in device with the excellent cyclic response-recovery behavior exhibited the high sensing capability and reproducibility of the GO-based device for humidity sensing. Meanwhile, the remarkable capacitance shift in our humidity sensory system may be mainly attributed to an ionic conductivity from the adsorption of water molecules with high dielectric constant (~80) at room temperature [67]. The cyclic absorption-desorption process of water molecules was schematically illustrated in Figure 6e. In detail, water molecules were primarily physisorbed on the surface of the partially reduced GO at low RH. In this case, during the course of humidity sensing process, the electrons generated from the hydrogen bonding between the oxygen-containing functional groups of the GO film and the water molecules applied, caused a capacitance shift and hence performed an important role in humidity sensors. However, with the increased moisture exposure to the active sites at high RH, water molecules were secondary-physisorbed with the intercalated water molecules inside of the GO film interface forming single hydrogen bonding [68,69]. In the irregular and discontinuous networks generated by hydrogen bonding of adjacent water molecules, the protons (H^+^) hopping between adjacent water molecules and migration in the form of hydronium (H_3_O^+^) ions act as charge carriers by Grotthuss mechanisms: (H_2_O + H_3_O→H_3_O + H_2_O) and contribute to the charge-transport [70]. Indeed, the water molecules intercalated into the multilayered structure of GO film lead to swelling effect, resulting in an increase of interlayer distance [71]. The increased interlayer distance of GO sheets by water molecules may cause a change in the multilayer GO structure as well as a decrease in the conductivity. Consequently, the swelling effect exposed humidity and ionic conductivity is a trade-off in multilayered GO film based humidity sensory systems.

## 4. Conclusions

In summary, we developed a facile deposition process of GO on flexible polymer substrates to create highly uniform ultrathin films over a large area using a FESA strategy. The prepared GO thin films could be selectively reduced by the simple laser-scribing process (i.e., LDW) for the programmable circuit printing with a desired configuration and high-performance humidity sensor arrays. By careful tuning of the laser-scribing operation, GO films were instantly converted to patterned rGO films, with a goal of lower sample damage due to the non-contact mode and the clear selectivity. Thus, the direct laser-scribing operation yielded the efficient reduction of GO film with high electrical stability and conductivity, as demonstrated by the interconnections for a packaged LED chip at a moderately applied voltage. In addition, partially reduced GO film prepared via indirect laser-scribing operation, bearing relatively abundant oxygen groups, was used for the humidity sensing application. The flexible capacitive humidity sensor which was designed to be attached to a fingernail demonstrated clear capacitance-fluctuation on the adsorption and desorption processes between the interface of water molecules and the oxygen-containing groups in the GO-basal plane. The cyclic characteristics of the response-recovery by varying the moisture exposure have been evaluated. Therefore, the excellent humidity sensing features may represent advanced topics for fundamental and practical works. Moreover, the extremely simple and one-step laser-scribing method for producing a single-layer printed circuit board with graphene-based traces or pads has other possible advantages for the implementation of functional multi-components associated with a commonly used SMT manufacturing process. We envision that the ability of biocompatible graphene-based materials may provide opportunities for extended applications, such as implantable electrodes or cell-culture platforms for the development of tissue engineering in the near future [72,73,74,75,76].

## Supplementary Materials

Supplementary materials can be found at http://www.mdpi.com/1424-8220/18/6/1857/s1, Figure S1: OM image of highly uniform GO thin film (~40 nm) on APTES-treated PP substrate produced by FESA process, Figure S2: Specification of laser system, Figure S3: OM images of the stripe-patterned rGO with the line width of ~180 μm by direct (left side) and indirect laser exposure (right side) under the condition of laser power 0.8 W and laser speed 100 mm/s; (a,b): patterns of overlapping lines, (c,d): line intervals of ~50 μm and (e,f): line intervals of ~200 μm, Figure S4: SEM images revealing the surface morphology of rGO film by indirect laser exposure through the PP barrier under the condition of laser power 0.8 W and laser speed 100 mm/s. (a) GO surface inside the indirectly laser-exposed region. (b) The boundary between laser exposed rGO region and unscribed region in GO thin film marked by the white dotted line. (c,d) highly magnified SEM images of rGO surface with surface craters and swollen structures formed by PP barrier at laser exposure, Figure S5: Representative AFM image of the noticeable interface differences between the laser-scribed GO region and unscribed GO region; Green line shows relative height profile (up to ~1.5 μm) to the left and right area, bounding the white dotted line.
