# Supplementary material for: One-Step Laser Patterned Highly Uniform Reduced Graphene Oxide Thin Films for Circuit-Enabled Tattoo and Flexible Humidity Sensor Application
Source: Sensors (Basel). 2018 Jun 6;18(6):1857. doi: 10.3390/s18061857 (PMC6021857; doi:10.3390/s18061857)
Supplement: Supplementary file 1 [file sensors-18-01857-s001.pdf]

sensors

Supporting Information

One-Step Laser Patterned Highly Uniform Reduced Graphene Oxide Thin Films for Circuit-enabled Tattoo and Flexible Humidity Sensor Application

Rowoon Park[1,†], Hyesu Kim[1,†], Saifullah Lone[1,†], Sangheon Jeon[1], Young Woo Kwon[2], Bosung Shin[1,]* and Suck Won Hong[1,]*

[1] Department of Cogno-Mechatronics Engineering, Department of Optics and Mechatronics Engineering, College of Nanoscience and Nanotechnology, Pusan National University, Busan 46241, Republic of Korea.

[2] Department of Nano-Fusion Technology, College of Nanoscience and Nanotechnology, Pusan National University, Busan 46241, Republic of Korea.

* Correspondence: bosung@pusan.ac.kr (B.S.); swhong@pusan.ac.kr (S.W.H.)

† These authors contributed equally to this work.

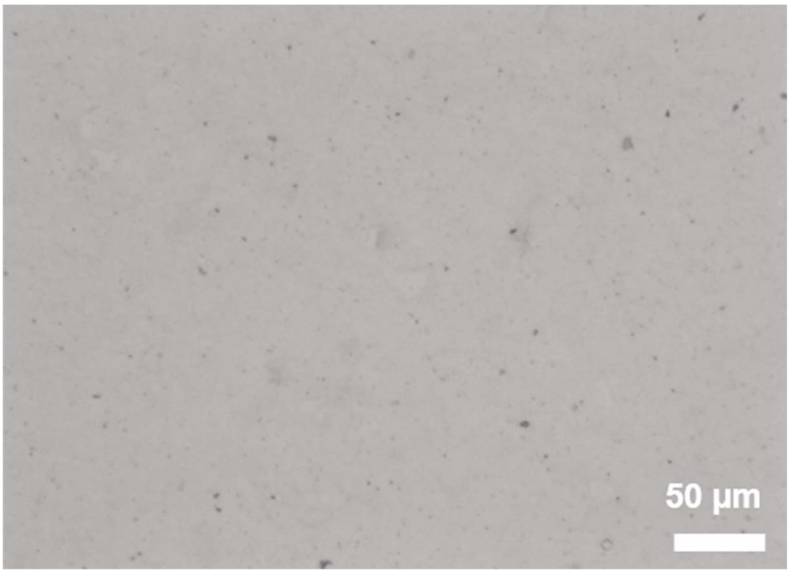

Figure S1. OM image of highly uniform GO thin film (~40 nm) on APTES-treated PP substrate produced by FESA process.

Parameter	Value
**Source**	Nd:YVO4 UV pulsed laser
**Wavelength (nm)**	355
**Average power (W)**	0.8
**Pulse length (ns)**	20
**Repetition rate (kHz)**	30
**Beam diameter (mm)**	1.5
**Beam divergence (mrad)**	< 0.5
**Beam Mode (m^2)**	< 1.25 TEM_{00}

Figure S2. Specification of laser system.

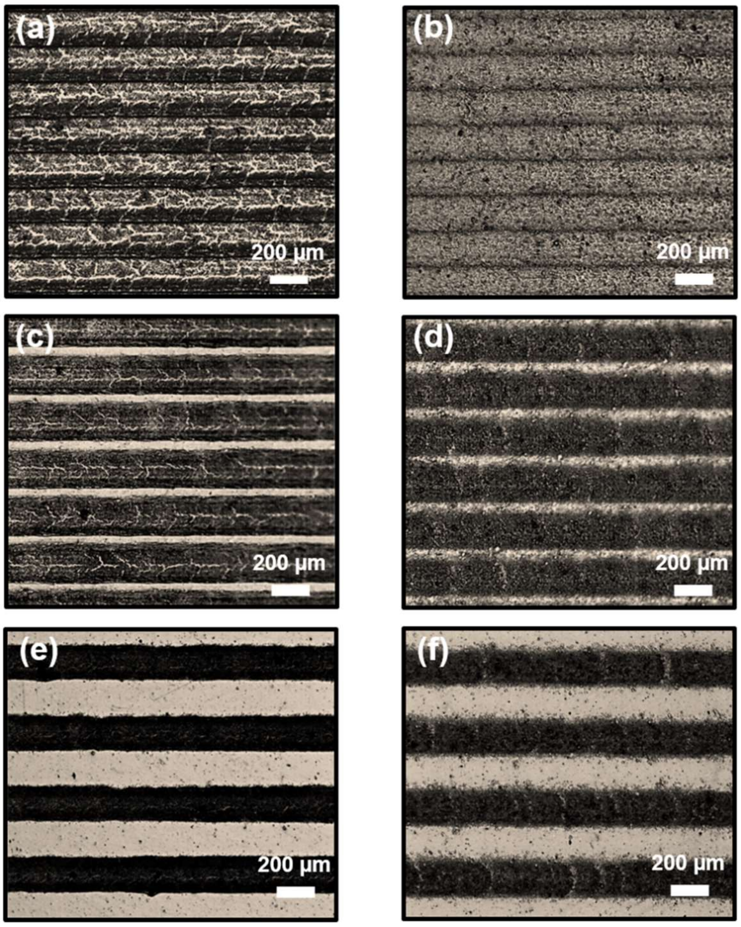

Figure S3. OM images of the stripe-patterned rGO with the line width of ~180 µm by direct (left side) and indirect laser exposure (right side) under the condition of laser power 0.8 W and laser speed 100 mm/s; (a), (b):patterns of overlapping lines, (c), (d):line intervals of ~50 µm and (e), (f):line intervals of ~200 µm.

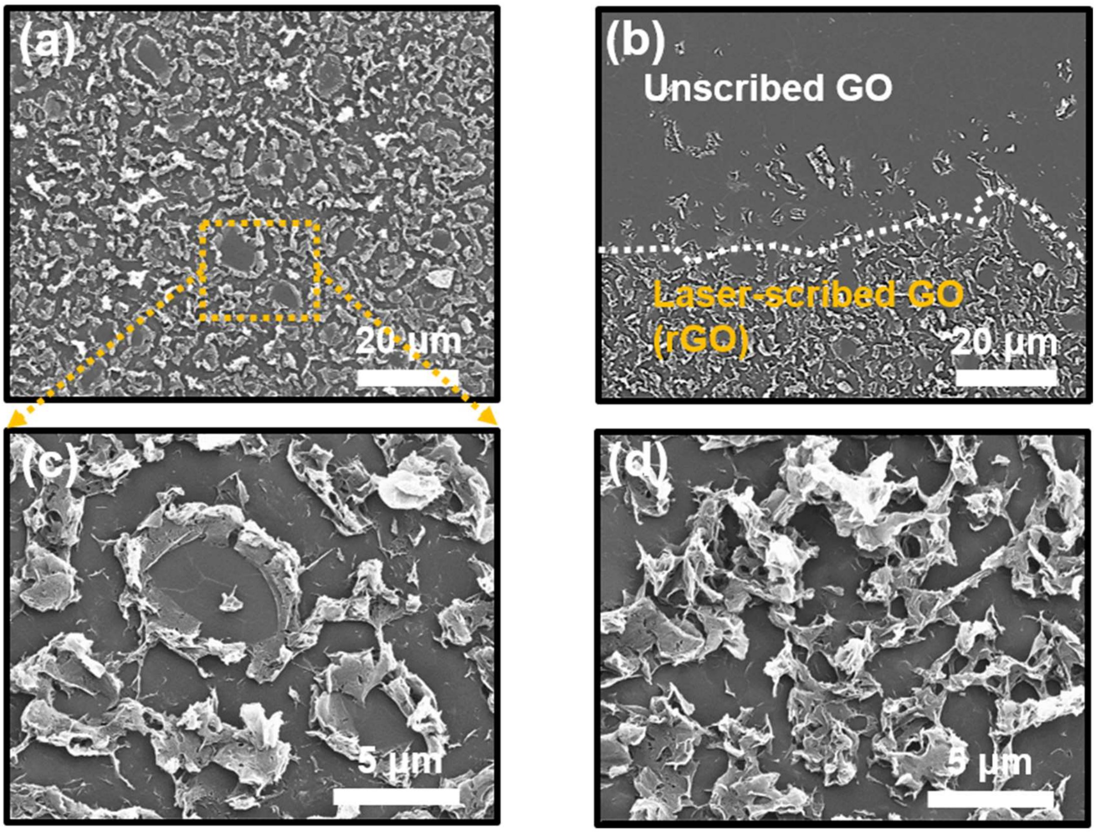

Figure S4. SEM images revealing the surface morphology of rGO film by indirect laser exposure through the PP barrier under the condition of laser power 0.8 W and laser speed 100 mm/s. (a) GO surface inside the indirectly laser-exposed region. (b) The boundary between laser exposed rGO region and unscribed region in GO thin film marked by the white dotted line. (c) and (d) highly magnified SEM images of rGO surface with surface craters and swollen structures formed by PP barrier at laser exposure.

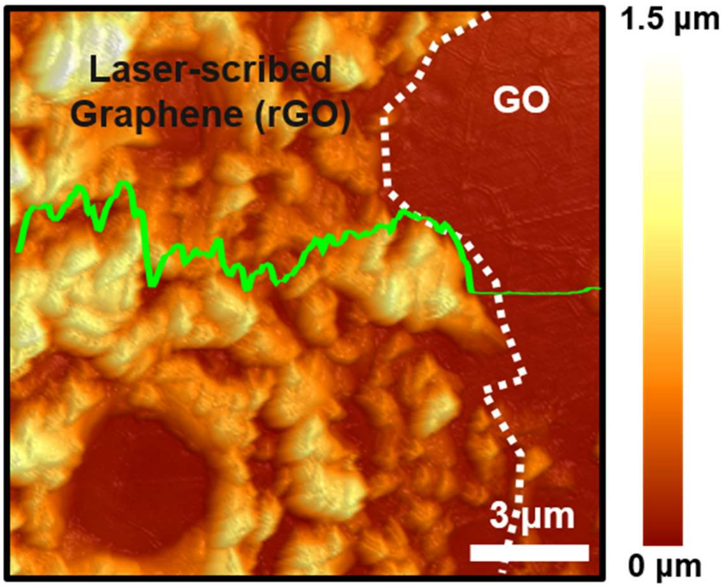

Figure S5. Representative AFM image of the noticeable interface differences between the laser-scribed GO region and unscribed GO region; Green line shows relative height profile (up to ~1.5 μm) to the left and right area, bounding the white dotted line.

© 2018 by the authors. Submitted for possible open access publication under the terms and conditions of the Creative Commons Attribution (CC BY) license (http://creativecommons.org/licenses/by/4.0/).
